# Antidepressant-like Effects of *Orthosiphon stamineus*: Integrated Evidence from In Silico, In Vitro, and a Reserpine-Induced Depression Model

**DOI:** 10.3390/molecules31162892

**Published:** 2026-08-19

**Authors:** Yousaf Dawood, Atheer Zgair, Mun Fei Yam, Nur Hidayah Kaz Abdul Aziz

**Affiliations:** 1 School of Pharmaceutical Sciences, Universiti Sains Malaysia, George Town 11800, Penang, Malaysiayammunfei@usm.my (M.F.Y.); 2 College of Pharmacy, University of Anbar, Ramadi 31001, Iraq

**Keywords:** molecular docking, antidepressants, MAO-A, behavioral study, BDNF, immunohistochemistry

## Abstract

Background: Traditional antidepressants are associated with undesirable side effects, limited efficacy, and delayed therapeutic responses. Natural products have emerged as a potential alternative to these drugs. This research aimed to investigate the antidepressant-like activity and possible mechanism of *Orthosiphon stamineus* (OS) leaf ethanolic extract. Methods: Molecular docking and in vitro studies were performed to assess the inhibitory activity of ethanolic extracts of OS leaves against the monoamine oxidase (MAO-A) enzyme. Behavioral tests were used to evaluate the antidepressant-like activity of the 95% ethanol extract in reserpine-treated rats, followed by histopathological examination and measurement of hippocampal serotonin (5-HT), norepinephrine (NE), and brain-derived neurotrophic factor (BDNF). Results: The main active compounds, particularly rosmarinic acid and eupatorin, exhibited favorable binding affinities to the MAO-A active site. The 95% and 50% ethanol extracts of OS showed dose-dependent MAO-A inhibitory activity with ICs50 of 88.67 and 103.2 µg/mL, respectively. The 95% ethanol extract of OS leaves showed a significant reduction in immobility time, restored locomotor activity, and preserved hippocampal morphology. Notably, the higher dose of OS extract (150 mg/kg) significantly enhanced hippocampal expression of 5-HT and NE, with a moderate upregulation of BDNF. Conclusions: These findings suggest that the 95% ethanol extract of OS leaves exhibits promising antidepressant-like effects, which may be mediated through monoamine modulation and BDNF expression and partially through MAO-A enzyme inhibition.

## 1. Introduction

Depression is a neuropsychiatric condition expected to be one of the leading causes of disability worldwide by 2030 [1]. Treatment with conventional antidepressants, particularly monoamine oxidase enzyme inhibitors and selective serotonin reuptake inhibitors, is often limited by adverse effects, suboptimal efficacy, and delayed therapeutic response [2]. These limitations underscore the urgent need for novel antidepressant agents with improved safety and efficacy. Herbal products are one of the most promising approaches that have recently received great interest. These products provide an advantage, as they exhibit a favorable safety profile compared with conventional medications. In recent years, there has been growing interest in herbal and plant-derived therapeutics for neuropsychiatric disorders due to their pharmacological versatility and relatively low toxicity. *Orthosiphon stamineus* (OS) leaf is a medicinal herb traditionally known as cat’s whiskers or Java tea. It is commonly used by Asian people as a diuretic and for urinary tract infection. Several studies established numerous therapeutic applications of OS, such as the treatment of bacterial and viral infections, inflammation, rheumatism, jaundice, and cancer-related conditions, including angiogenesis [3]. The diverse pharmacological properties of OS leaves have been attributed to its wide content of phytochemical compounds involving phenolic compounds (flavonoids and phenolic acids), terpenoids, and other volatile constituents contained in its essential oil [4]. The most important pharmacologically active compounds identified in OS leaf are eupatorin (EUP), rosmarinic acid (RA), sinensetin (SEN), and 3-hydroxy-5,6,7,4-tetramethoxyflavone (TMF) [5]. Our previous HPLC analysis identified a lower concentration of RA and EUP in the aqueous extract of OS leaves, whereas SEN and TMF were not detected under the experimental conditions used [6]. Based on this, the ethanol extracts were selected for further investigation, whereas the aqueous extract was excluded from the present study. Although OS leaves have been extensively studied for its anti-inflammatory, antimicrobial, and hepatoprotective effects, limited data are available on its potential neuroprotective or antidepressant effects. Retinasamy and collaborators demonstrated that ethanolic extract of the OS leaves can improve memory in scopolamine-induced memory dysfunction in animal models, indicating that the OS plant has a protective effect against neurodegenerative diseases such as Alzheimer’s disease [7]. Additionally, Rosmarinic acid (RA), the major pharmacologically active compound of OS, was shown to possess anxiolytic and antidepressant effects, which are attributed, at least in part, to its antioxidant and anti-inflammatory properties [8]. From these findings, it is hypothesized that the OS leaf extract may likewise possess antidepressant-like activity. Therefore, the present study aimed to evaluate the antidepressant-like effects of OS leaf ethanolic extract and elucidate its potential underlying mechanisms that may contribute to its pharmacological activity.

## 2. Results

### 2.1. Molecular Docking Analysis

A total of four selected compounds from OS (EUP, RA, SEN, and TMF) have been successfully docked against the crystal structure of the MAO-A enzyme. These compounds displayed binding energies against the MAO-A (PDB ID: 2bxr) ranging between −8.03 and −9.16 kcal ${\mathrm{mol}}^{-1}$, compared with −6.92 kcal ${\mathrm{mol}}^{-1}$ for the co-crystallized MAO-A inhibitor, clorgyline, as shown in Supplementary Data Table S1. These computational scores suggest that all selected compounds exhibited favorable predicted accommodation within the MAO-A active site. However, these results do not imply that they have a higher binding affinity or inhibition potency than clorgyline. In its predicted binding pose, clorgyline exhibited several hydrophobic (π–alkyl and π–sigma) interactions with flavin adenine dinucleotide (FAD), Tyr444, Tyr407, Tyr69, and Leu337 and formed a π-π interaction with the Phe208 residue. Nevertheless, the selected compounds formed H-bond interactions in addition to several hydrophobic contacts involving the FAD cofactor and key MAO-A active residues. EUP formed two hydrogen bonds with Tyr444 and Thr336 and further engaged in π–alkyl and π–sigma interactions with the FAD cofactor, Ile335 and Tyr407, in addition to several π-π stacking interactions with Phe208 and Tyr444. On the other hand, RA established a remarkably binding profile, forming five hydrogen bond interactions with key residues within the active site, Tyr444, Arg206, and Ser209; in addition, it formed several π-π stacking interactions with Tyr69, Phe208, Trp441, and Gly7. However, RA does not directly contact the FAD cofactor; instead, Tyr69 acts as a connecting residue mediating their interaction. SEN displayed classical hydrogen interaction with the Ser209 residue; in addition, it formed several π-π stacking and π–alkyl interactions with the FAD cofactor, Tyr407, Phe208, Tyr444, Phe209, Leu337, and IleE335. TMF showed three hydrogen bonds with Pro72, Thr73, and Arg206, in addition to many π–alkyl and π–sigma interactions with FAD, Tyr444, Tyr69, Phe352, Phe208, Pro72, and Arg206, and further established π-π stacking interactions with Trp441. Three-dimensional (3D) and two-dimensional (2D) illustrations of compound configurations at the binding pocket of the MAO-A enzyme are illustrated in Supplementary Data (Figure S1).

### 2.2. In Vitro MAO-A Inhibition Assay

**Figure 1:**
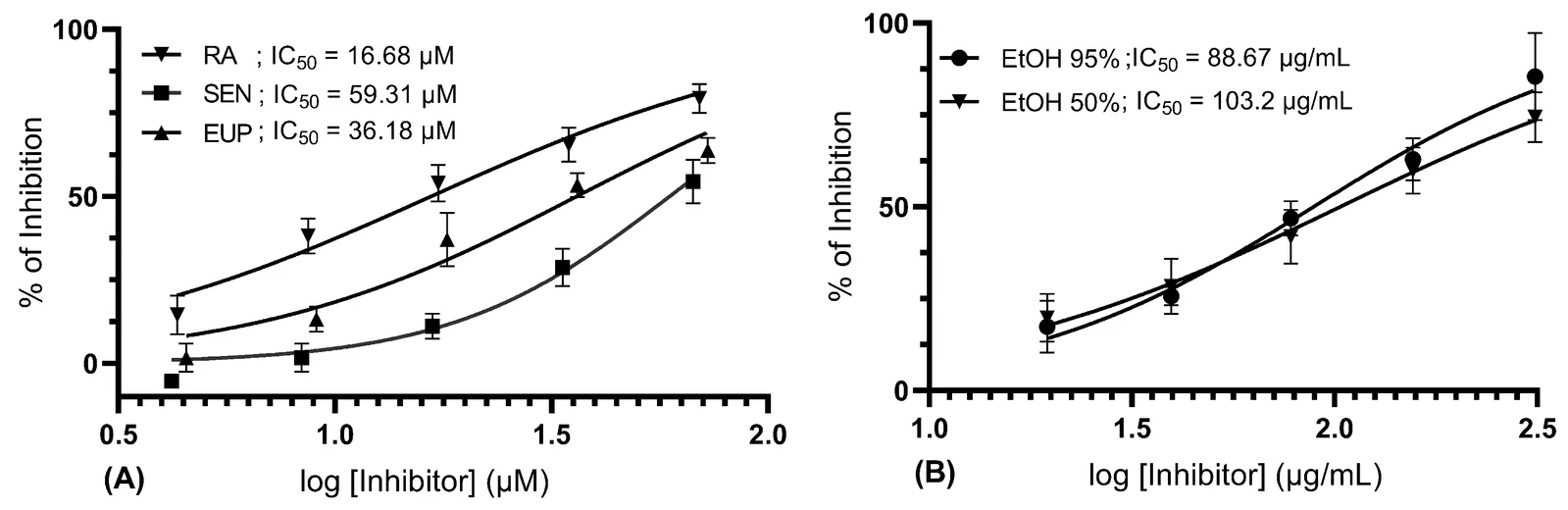
MAO-A enzyme inhibition dose–response curve for (**A**) Pharmacologically active compounds (RA, rosmarinic acid; SEN, sinensetin; and EUP, eupatorin) and (**B**) Ethanol extract of *Orthosiphon stamineus* leaves (EtOH 95% and 50%). The data are presented as the mean ± SD of three replicates of each concentration (*n* = 3). IC50 values were calculated using non-linear regression (4-parameter logistic curve).

Monoamine oxidase enzymes are responsible for the degradation of serotonin, dopamine, and norepinephrine in the central nervous system. In this study, 50% and 95% ethanol extracts of OS leaves, EUP, RA, SEN, and the positive control inhibitor, clorgyline, were used to examine the inhibitory effects toward the MAO-A enzyme. All tested compounds showed a dose-dependent inhibitory pattern. RA exhibited a significant inhibition effect (38.17%, p< 0.0001) at a concentration of 8.66μM and displayed the lowest IC50 among all tested compounds: 16.68 ± 1.4μM. On the other hand, EUP and SEN showed higher IC50 values (36.18 ± 3.66 and 59.31 ± 4.08μM, respectively); however, they are still effective at higher concentrations (Figure 1A). In contrast, clorgyline exhibited 92% inhibition relative to the control. The 95% ethanol extract exhibited a higher inhibitory effect than the 50% extract. The former showed an IC50 value of 88.67 ± 7.6, whereas the IC50 value exhibited by the 50% extract was 103.20 ± 9.70 µg/mL (Figure 1B). Nevertheless, at 39.06 µg/mL, the 50% extract achieved significant inhibition with a percentage higher than that exhibited by the 95% extract at the same concentration (28.40% p< 0.01 and 22.15% p< 0.05, respectively).

### 2.3. The Effect of OS Leaf Ethanolic Extract on Body Weight

**Figure 2:**
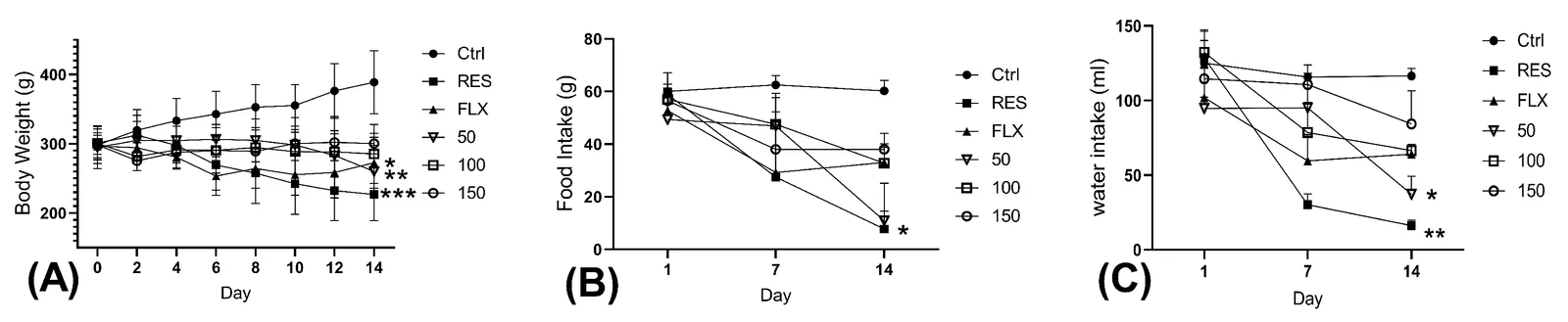
Effect of the 95% ethanol extract of *Orthosiphon stamineus* (OS) leaves (50, 100, and 150 mg/kg) and fluoxetine (FLX) on physiological parameters in reserpine (RES)-induced depressive rats: (**A**) body weight, (**B**) food intake, and (**C**) water intake over 14 days of treatment. All results are represented as the mean ± S.E.M. (*n* = 7); two-way ANOVA; * p< 0.05; ** p< 0.01; *** p< 0.001 vs. control (Ctrl).

At the beginning of the experiment, the weights of the rats were approximately equivalent, and no significant differences were observed. After 14 days of treatment, the control group exhibited weight gain, while reserpine caused significant weight reduction compared with the control group. However, fluoxetine-treated rats established a slight reduction in body weight during the first 10 days of treatment but regained their weight at the end of the experiment. Animals treated with the 95% ethanol extract of OS leaves showed dose-dependent responses; the low-dose (50 mg/kg) group exhibited significant weight loss over time compared with the control group, while those receiving 150 mg/kg showed slight weight changes throughout the treatment period (Figure 2A).

### 2.4. The Effect of OS Leaf Ethanolic Extract on Food and Water Intake

Measurement of daily consumption of food and water is an important indicator of the appetite behavior of the animals. This study showed no changes in food and water consumption after the first day of treatment. On day 7, a significant decline in water intake was observed (p< 0.05) in the reserpine-treated group. On day 14, reserpine significantly reduced food (p< 0.05) and water intake (p< 0.01) compared with the control group. Similarly, rats treated with OS leaf ethanolic extract at 50 mg/kg showed a significant decrease in water consumption (p< 0.05) and a non-significant reduction in food intake. However, rats treated with higher doses of OS extract (100 and 150 mg/kg) and fluoxetine did not show significant changes in food intake and water consumption relative to the control group (Figure 2B and Figure 2C, respectively).

### 2.5. The Effect of OS Leaf Ethanolic Extract on Animal Behavior

#### 2.5.1. Forced Swimming Test (FST)

**Figure 3:**
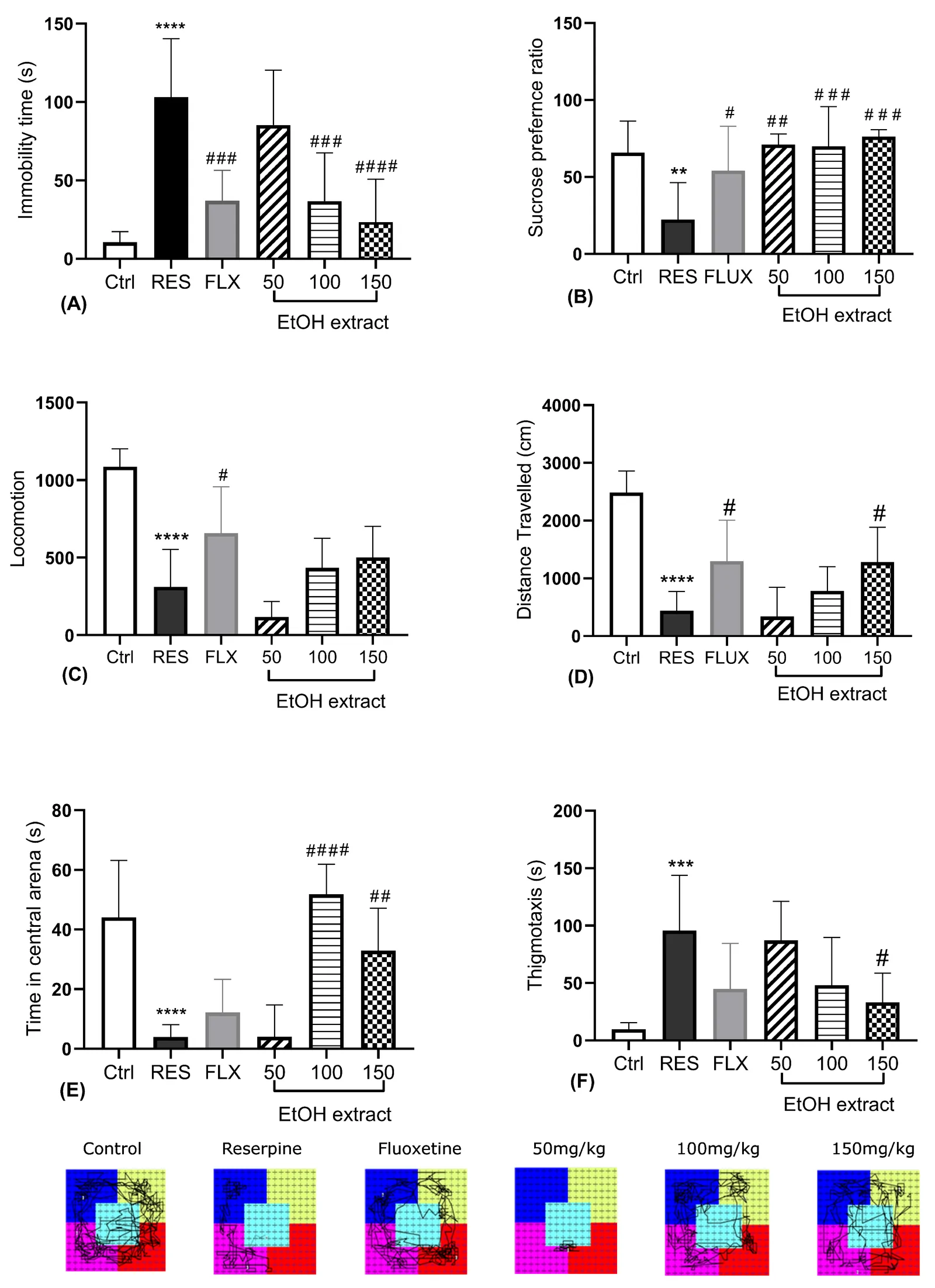
Evaluation of rat’s behavior following treatment with different doses of the 95% ethanol extract of *Orthosiphon stamineus* (OS) leaves (50, 100, and 150 mg/kg) and fluoxetine (Flux): (**A**) Immobility time, (**B**) Sucrose preference test, (**C**) Locomotion, (**D**) Distance traveled, (**E**) Time in the central arena, and (**F**) Thigmotaxis. All results are represented as means ± S.E.M. (*n* = 7); one-way ANOVA; ** p< 0.01, *** p< 0.001 and **** p< 0.0001 vs. control; # p<0.05, ## p<0.01, ### p<0.001, and #### p<0.0001 vs. the reserpine (RES) group.

FST is used to measure depressive-like symptoms in rodents according to the supposition that animal despair represented by immobility is an indicator of anhedonia. Figure 3A illustrates the FST results; reserpine increased the immobility time significantly (99 ± 18.38 s, p< 0.001) compared with the control group (10.57 ± 2.57 s), indicating depressive-like behavior. Treatment with the 95% ethanol extract of OS leaves at 50 mg/kg did not significantly reverse this effect. However, fluoxetine and the high doses of the extract (100 and 150 mg/kg) displayed a significant decrease in the immobility time when compared with the reserpine-administered group (38.86 ± 15.10 s, p< 0.05; 32 ± 11.66 sec, p< 0.05; 23.29 ± 10.40 s, p< 0.01, respectively).

#### 2.5.2. Sucrose Preference Test (SPT)

Rodents intuitively like sweet food or solutions, and a reduction in the preference for sweet food or solutions means a decrease in the ability of these animals to experience pleasure. Therefore, SPT is considered as an indicator of loss of interest or anhedonia. Figure 3B illustrates the SPT results in which reserpine significantly reduced sucrose preference in stressed animals (33.41 ± 13.48, p< 0.01) compared with untreated control rats (65.78 ± 7.74). However, this reduction was significantly attenuated by administering fluoxetine and the OS leaf ethanolic extract. Specifically, fluoxetine improved sucrose preference by approximately 20% (54.14 ± 10.90, p< 0.05). On the other hand, repeated administration of the OS extract at 50, 100, and 150 mg/kg improved sucrose preference by 60.93 ± 10.42, p< 0.01; 69.94 ± 9.72, p< 0.001; and 76.33 ± 1.64, p< 0.001, respectively, compared to the reserpine group.

#### 2.5.3. Open Field Test (OFT)

This study examined the effect of the 95% ethanol extract of OS leaves on locomotor activity in reserpine-induced depressive rats. The open field tracking system showed that reserpine reduced locomotor activity and total distance traveled compared with the control group (310.67 ± 141 cm, p< 0.0001; and 441.82 ± 135 cm, p< 0.0001, respectively). Fluoxetine treatment improved both locomotion and distance traveled significantly compared with the reserpine group (658 ± 131 cm, p< 0.05; and 1299 ± 312.63 cm, p< 0.05, respectively). Treatment with the OS extract showed dose-dependent improvement in both locomotion and distance traveled (Figure 3C and Figure 3D, respectively); however, only the 150 mg/kg dose exhibited a significant increase in total distance traveled (1016 ± 201.4 cm, p< 0.05) compared to the reserpine group. Regarding the time spent by the animals exploring the central arena, as shown in Figure 3E, reserpine exhibited a significant reduction in the time spent in the central arena. However, rats treated with OS extract at 100 and 150 mg/kg showed an obvious increase in the time spent by animals in the central arena. Alternatively, reserpine significantly increased the time spent by the animals near the walls (95.7 ± 19.5 s, p< 0.001) compared with the control group (9.7 ± 2.3 s). In contrast, OS extract at 150 mg/kg significantly reduced thigmotaxis (33.1 ± 11.3 s, p< 0.05) in reserpine-treated animals; however, no significant changes were observed by fluoxetine and the 50 and 100 mg/kg doses of OS extract compared with the reserpine group (Figure 3F).

### 2.6. Quantification of NE, 5-HT, and BDNF by ELISA

**Figure 4:**
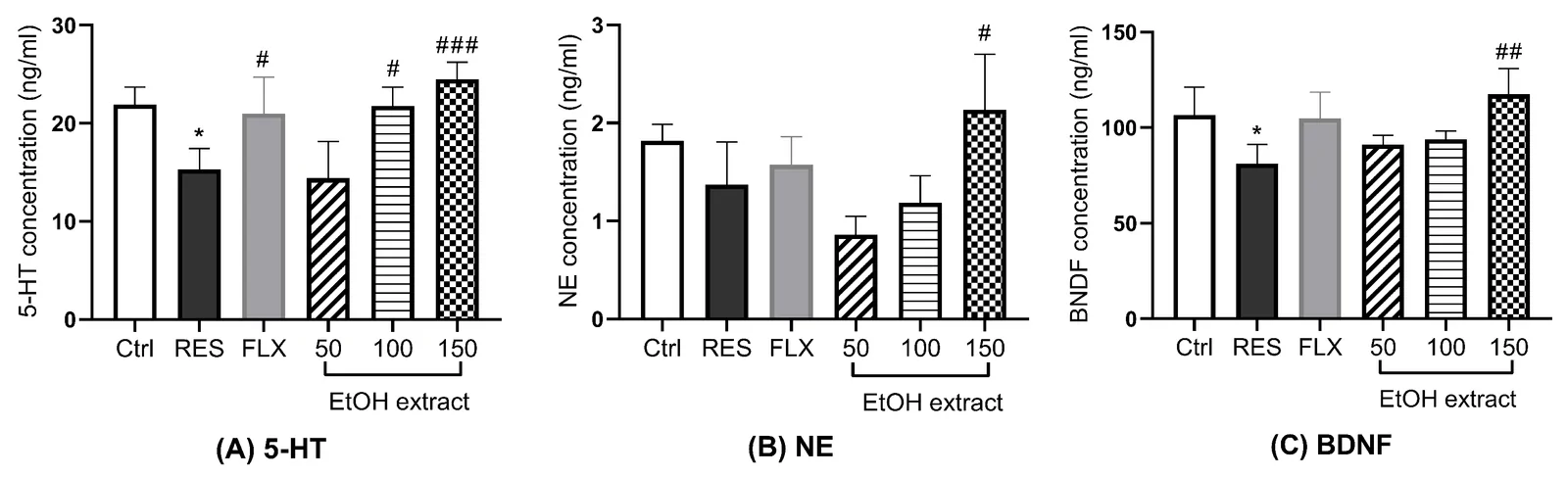
Hippocampal neurochemical changes following the 95% ethanol extract of *Orthosiphon stamineus* (OS) leaves (50, 100, and 150 mg/kg) and fluoxetine treatment. (**A**) 5-HT, (**B**) NE, and (**C**) BDNF levels were measured via ELISA. Values are presented as means ± S.E.M. (*n* = 4); one-way ANOVA; * p< 0.05 vs. control; # p< 0.05; ## p< 0.01; ### p< 0.001 vs. reserpine.

The effect of the 95% ethanol extract of OS leaves on the brain markers NE, 5-HT, and BDNF is illustrated in Figure 4. Reserpine showed a significant reduction in 5-HT content (15.30 ± 1.06 ng/mL, p< 0.05) compared with the control group. The 50 mg/kg dose of OS extract did not reverse reserpine-induced 5-HT depletion. In contrast, fluoxetine and OS extract at 100 and 150 mg/kg increased 5-HT concentration in the hippocampus significantly (21 ± 1.85 ng/mL; 21.77 ± 0.96 ng/mL, p< 0.05; and 24.5 ± 0.87 ng/mL, p< 0.001, respectively) compared with the reserpine group (Figure 4A). Regarding NE, reserpine did not decrease the NE in the hippocampus significantly relative to the control group. Similarly, fluoxetine and the OS extract at doses 50 and 100 mg/kg did not significantly alter NE levels compared to reserpine-treated animals. However, OS extract at 150 mg/kg substantially increased NE concentration after 14 days of treatment (2.13 ± 0.28 ng/mL, p< 0.05) (Figure 4B). BDNF levels were significantly reduced in the reserpine-treated group compared with the control group (p< 0.01). All treatment groups, including fluoxetine and OS extract groups, showed increased BDNF levels; however, only the 150 mg/kg extract group exhibited a significant increase (117.47 ± 6.73 ng/mL, p< 0.01) compared with reserpine-treated animals (Figure 4C).

### 2.7. The Effect of OS Extract on Hippocampal Morphology

**Figure 5:**
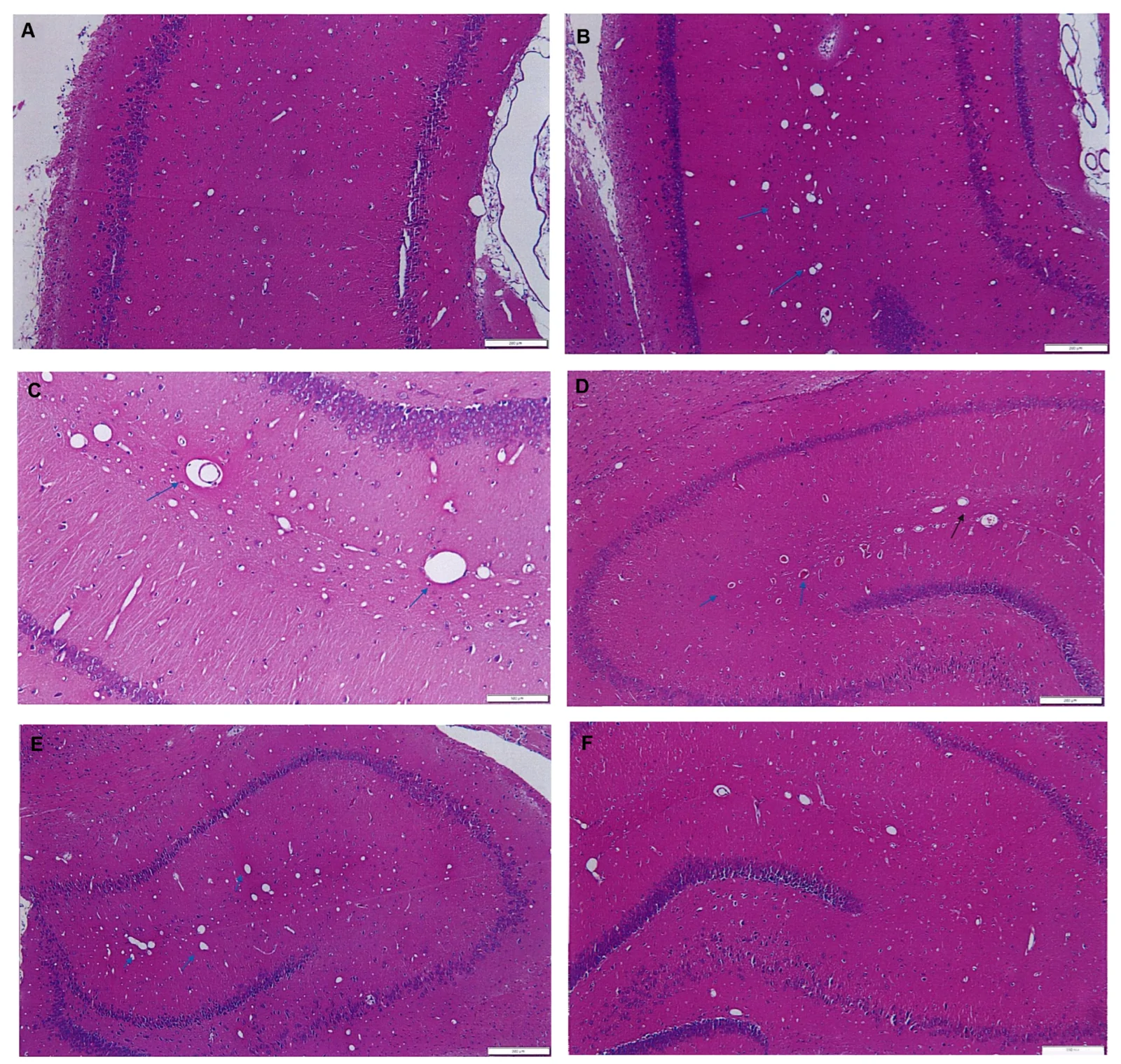
Histological evaluation of the hippocampus using H&E staining. (**A**) control, (**B**) reserpine, (**C**) fluoxetine, and 95% ethanol extract of *Orthosiphon stamineus* (OS) leaves at: (**D**) 50, (**E**) 100, and (**F**) 150 mg/kg. Bar scale 200 µM.

Hematoxylin & eosin staining outcomes of hippocampal tissues are represented in Figure 5. The hippocampal morphology in the control group appears normal, with preserved laminar organization. The neurons are arranged in a tightly packed layer consistent with pyramidal neurons. There is no evidence of pathological changes in the control group (Figure 5A). However, the reserpine-treated group showed a decrease in cellular density compared with the control tissue; additionally, the laminar organization was less defined. Mild vacuolation of neuropil was observed in the cornu ammonis (CA) area, which may indicate neuronal inflammation (arrow) (Figure 5B). In the fluoxetine group, vacuolation with eosinophilic neuropils was observed surrounding the area at the subiculum hilus area (arrow); these changes may indicate inflammatory reactions (Figure 5C). Rats treated with a low dose of OS leaf ethanolic extract (50 mg/kg) showed mild congestion of blood capillaries in the CA area and moderately reduced neuropil vacuolation compared to the reserpine group (Figure 5D). Similarly, mild vacuolation of the neuropil was observed in the CA region of the hippocampus tissue of rats treated with OS extract 100 mg/kg (Figure 5E). In contrast, a high dose of OS leaf extract (150 mg/kg) did not show abnormal lesions in the hippocampus tissue, no vacuolation, and well-preserved lamination, indicating a potential neuroprotective effect (Figure 5F).

### 2.8. The Effect of OS Leaf Ethanolic Extract on Hippocampal Immunostaining

**Figure 6:**
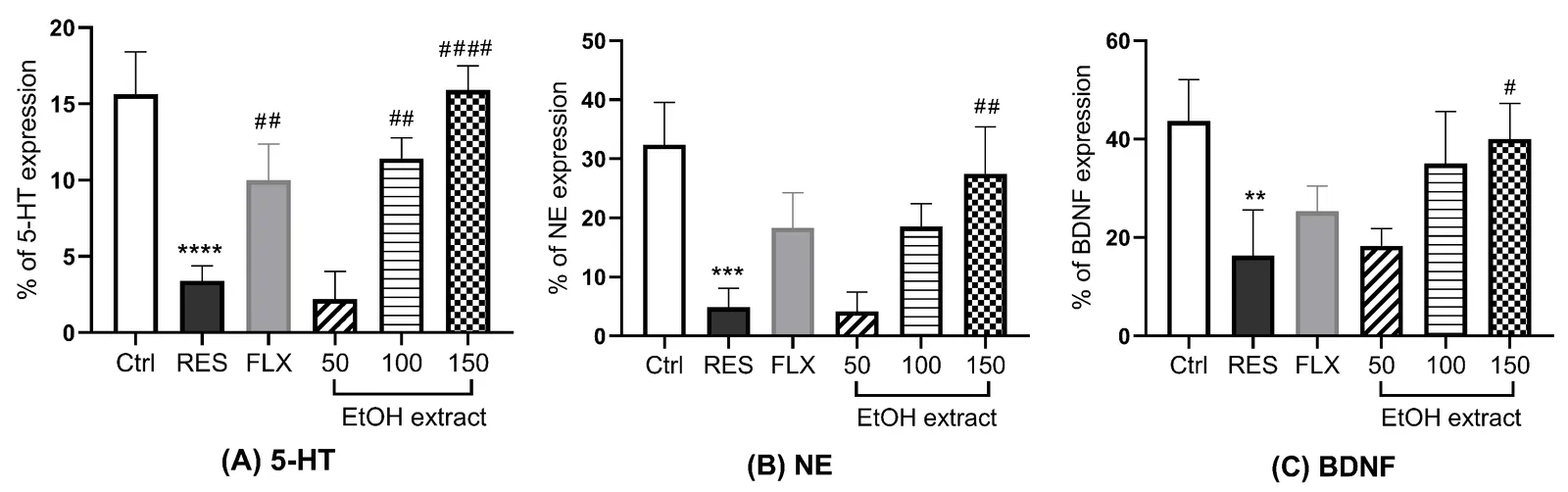
Quantitative immunohistochemical analysis of hippocampal neurochemical markers in reserpine-induced depressive rats treated with 95% ethanol extract of *Orthosiphon stamineus* (OS) leaves (50, 100, and 150 mg/kg) and fluoxetine treatment. (**A**) Percentage of 5-HT expression, (**B**) Percentage of NE expression, and (**C**) Percentage of BDNF expression. Values are expressed as means ± S.E.M. (*n* = 3); one-way ANOVA; ** p< 0.01; *** p< 0.001; and **** p< 0.0001 vs. control group; # p< 0.05; ## p< 0.01; and #### p< 0.0001 vs. the reserpine group.

In hippocampus sections, the calculated percentage of positive neurons in various subareas of the hippocampus, such as the dentate gyrus (DG) and cornu ammonis regions (CA1, CA2, and CA3), was expressed for three brain markers, 5-HT, NE, and BDNF, as shown in Figure 6A–C, respectively. Immunohistochemical analysis revealed significant reductions in the hippocampal immunoreactivity for all three markers—5-HT, NE, and BDNF—in rats treated with reserpine compared with the control group. Treatment with the 95% ethanol extract of OS leaves at 100 and 150 mg/kg resulted in a significant increase in the 5-HT biomarker (11.43 ± 0.78, p< 0.01 and 15.90 ± 0.91, p< 0.0001, respectively) compared with the reserpine-treated rats, which is comparable to the effect of fluoxetine. On the other hand, OS extract at a dose of 50 mg/kg showed a relatively low percentage of 5-HT-immunopositive neurons after 14 days of treatment. The intensity of 5-HT immunostaining in different animal groups is shown in Supplementary Data Figure S2a. Regarding NE expression, only the 150 mg/kg OS extract group showed a significant improvement (27.46 ± 4.6, p< 0.01) in NE expression compared to the reserpine group (Figure 6B), while fluoxetine and the 100 mg/kg OS extract group exhibited a non-significant trend towards increased NE labeling compared with the hipoocampal sections of reserpine-treated rats (Figure S2b). BDNF immunoreactivity followed a pattern similar to that shown for NE. The 150 mg/kg OS extract group exhibited the highest BDNF immunopositive neurons after 14 days of treatment (40 ± 4.16, p< 0.05) compared with the reserpin group (Figure 6C). Rats treated with 100 mg/kg OS extract and fluoxetine established a slight increase in BDNF immunopositive neurons, indicating a dose-dependent neuroprotective effect. Figure S2c shows immunostaining sections of the BDNF group. These findings suggest that the 95% ethanol extract of OS leaves modulates hippocampal neurochemical pathways involved in depression.

## 3. Discussion

This study provides evidence demonstrating the antidepressant-like effect of the 95% ethanol extract of OS leaves through comprehensive in silico, in vitro, and in vivo investigations. Molecular docking simulation demonstrated that the selected compounds showed favorable binding affinities toward the MAO-A enzyme and interact with the key residues associated with ligand recognition. However, these observations are only hypothesis-generating and lack functional inhibition or relative superiority over the reported MAO-A inhibitor, clorgyline. The effectiveness of enzyme inhibition depends on how ligands bind catalytically key active residues, determining the binding pose, duration, and functional impact. In this study, the selected active compounds of OS formed a network of hydrogen bonds and hydrophobic interactions with key active residues within the MAO-A enzyme, including Tyr69, Tyr407, Tyr444, Phe208, Arg206, Ser209, Trp441, and Ile335. For example, RA formed multiple hydrogen bonds with the active pocket of the binding site together with several π-π stacking interactions involving Phe208 and Tyr69. These interactions stabilize RA binding and elucidate its significant in vitro inhibitory effect, reflected by its lowest IC50 value. While SEN and EUP formed fewer hydrogen bonds, they exhibited considerable hydrophobic contacts with key residues, suggesting a complementary role of these interactions to overall binding stability. Several structural and mutational studies have demonstrated the crucial role of residues Tyr69, Tyr407, and Tyr444 in forming an aromatic “sandwich” that stabilizes bound ligands [9,10]. These residues promote substrate and inhibitor recognition; thus, their direct interaction with the active compounds of OS supports the potential effectiveness of these compounds as functional inhibitors.

Additionally, the FAD co-factor, which represents the distinctive component of MAO-A catalysis wherein redox reactions are carried out to achieve substrate oxidation, formed multiple hydrophobic interactions and π contacts with the selected compounds. Such interactions may interfere with FAD redox cycling and inhibit catalytic turnover. In contrast, clorgyline showed only hydrophobic interactions with the FAD moiety and lacked the hydrogen bonds that were detected with the selected compounds, which may clarify its comparatively favorable binding energy.

These computational findings were supported by in vitro MAO-A inhibition assays. Among the compounds investigated, RA showed the lowest IC50 (16.68μM), consistent with its extensive interactions within the active site, including FAD-associated residues. EUP and SEN showed higher IC50 values (36.18μM and 59.31μM, respectively), in agreement with their relatively lower hydrogen bonds but considerable hydrophobic contacts. The reported differences between the favorable binding energy of TMF and its exclusion from the current in vitro analysis due to its low abundance in OS leaf extracts emphasize that pharmacokinetics, bioavailability, and solubility must be considered alongside binding affinity. Furthermore, both 50% and 95% ethanol extracts of OS leaves exhibited dose-dependent inhibitory effects on the MAO-A activity. This finding may lead to the assumption that the inhibitory effect produced by ethanol extracts could be attributed to RA or may be due to the synergistic effect of RA and EUP. Our findings are consistent with a previous study demonstrating that RA is capable of inhibiting the MAO-A enzyme in vitro [11]. Notably, while previous work reported an IC50 = 50μM for RA, this study showed a lower IC50 of 16.68μM. In addition, the findings presented by Hase et al. [12] reinforced our results, as they exhibited that RA administration elevates brain monoamine levels, thereby signifying a potential therapeutic benefit in managing AD.

To validate these results in vivo, a reserpine-induced depression model was involved. This is a critical step because the in vitro outcome does not guarantee brain bioavailability or functional effects since it does not consider systemic metabolism, the blood–brain barrier (BBB), active efflux, and complex physiological tissue interactions, which, nevertheless, make it a crucial step.

Reserpine effectively challenges the monoaminergic system via inhibiting VMAT and thus depletes synaptic monoamines [13]. Therefore, the reserpine-induced depressive-like behavior model is well-suited for evaluating compounds that restore monoaminergic neurotransmission. However, this model fails to mimic the clinical heterogeneity of the disease but has managed to reproduce selected features of depression.

In the present study, three behavioral tests, including FST, SPT and OFT, were performed to measure the animal’s behavioral despair, anhedonia, and anxiety-like behaviors. The high doses of OS leaf ethanolic extract significantly alleviated depressive-like despair, reduced the anxiety, and restored the reward processing. These effects align with previous findings, where a flavonoid-rich extract showed antidepressant-like effects possibly via modulation of several neurotransmitters, such as noradrenergic, dopaminergic, and serotonergic [14,15]. Crucially, administration of the high dose of the 95% ethanolic extract of OS leaves increased the hippocampal 5-HT, and NE in reserpine-treated rats. The notable serotonergic enhancement at ≥100 mg/kg, as shown by both ELISA and immunohistochemistry, reflecting possibly MAO-A inhibition and additional serotonin reuptake modulation.

Although the ELISA assay did not detect significant NE depletion following administration of reserpine—possibly due to regional heterogeneity or compensatory mechanisms—the increased number of NE-positive neurons in the 150 mg/kg OS extract group suggests enhanced noradrenergic tone over time.

The significant enhancement in hippocampal BDNF proteins after a 150 mg/kg dose of OS leaf extract represents an important finding of this study. Reduced BDNF is associated with major depressive disorder and antidepressant resistance [16]. The antidepressant-induced increase in BDNF stimulates synaptic plasticity and neural proliferation, supporting long-term mood regulation. Thus, the neurotrophic effect of OS could partly contribute to its therapeutic activity and hippocampal protection.

This research reflected the state of knowledge regarding antidepressant action as simultaneously restoring monoamines and upregulating BDNF. It has been reported that monoamines possibly regulate BDNF through cAMP response element-binding protein (CREB) cascade signaling [17]. Despite this study not directly assessing this mechanism, it is a potential avenue to investigate in the future.

Moreover, this study showed that OS leaf ethanolic extract at 150 mg/kg markedly ameliorates hippocampal neuronal loss, vacuolation, and disrupted lamination produced by reserpine administration. These changes are consistent with reports of neuroinflammation and oxidative injury in depression [18].

Accordingly, this indicates that OS leaf ethanolic extract may also have adjunctive anti-neuroinflammatory and antioxidant properties concomitant to neurotransmitter modulation.

Rosmarinic acid and flavonoids in OS leaf ethanolic extract are well-documented antioxidants that could decrease microglial activation and oxidative stress [19]. This neuroprotection is of great importance in reversing the structural brain changes which follow a chronic depression and may help to decrease risks of future neurodegeneration.

Despite RA exhibiting MAO-A inhibition in vitro, its detection in the 95% ethanol extract of OS leaves does not establish that it was individually responsible for the antidepressant-like effects shown in vivo. RA is abundant in numerous medicinal plants, and the biological effect of an herbal extract is determined by its concentration, availability, and general chemical composition. Hence, the present study applies specifically to the tested 95% ethanol extract of OS leaves and should not be extrapolated to other RA-containing herbs.

The biochemical and behavioral findings should be attributed to the 95% ethanol extract of OS leaves as a whole rather than a specific compound. Despite the active compounds (RA, EUP, SEN, and TMF) in this study being chosen based on their identification in the ethanol extracts and pharmacological characterstics, they represent only part of the OS leaf chemical composition. Other compounds, including flavonoids, phenolic acids, and terpenoids, may also contribute to the observed effects. Since bioassay-guided fractionation and individual administration of the selected compounds were not conducted in this study, the contribution of each compound and the existence of synergy can only be hypothesized.

Several limitations of this study should be acknowledged. The OS leaves were not verified by a taxonomist, and no voucher specimen was deposited. However, they were sourced from an established supplier recognized by Universiti Sains Malaysia. Additionally, the reserpine model does not include psychosocial stressors or genetic predispositions, which represent the principal characteristic of depression in humans. Furthermore, this study did not elucidate the signaling pathways involving OS active compounds with TrkB activation or CREB phosphorylation.

## 4. Materialsand Methods

### 4.1. Animals

Male Sprague–Dawley rats (weighing 200–300 g; 8 weeks old) were purchased from the Advanced Medical and Dental Institute, Universiti Sains Malaysia. The animals were kept (34 rats per cage) under standard conditions at a controlled temperature (23 °C) and a 12:12 h light/dark cycle, and we measured the amount of food/water ad libitum.

### 4.2. Plant Extraction and Phytochemical Identification

The extraction process and phytochemical identification and quantification have been reported in our previous study [6]. Briefly, fresh leaves of OS were sourced and dried by Herbagus Trading (Kepala Batas, Pulau Pinang, Malaysia). The ground leaves (100 g) were extracted repeatedly with ethanol (95% and 50%) or water using heated maceration at 60 °C. The resulting extracts were concentrated using a rotary evaporator and stored at −4 °C until further use. The same extract preparation was used in the present study.

### 4.3. Chemicals

Rosmarinic acid (RA; Cat no. R4033; ≥98%), sinensetin (Sen; Cat no. SML1787; ≥98%), eupatorin (EUP; Cat no. E4660; ≥98%), and 3-hydroxy-5,6,7,4-tetramethoxyflavone (TMF; Cat no. CDS007351; ≥98%), originally purchased from Sigma-Aldrich (St. Louis, MO, USA), were kindly donated by Dr. Yam Mun Fei, School of Pharmaceutical Sciences, USM. The MAO-A Inhibitor Screening Kit (Fluorometric) was bought from Abcam (Cambridge, UK). Reserpine and fluoxetine hydrochloride were purchased from Tokyo Chemical Inc. (Tokyo, Japan).

### 4.4. Molecular Docking Study

#### 4.4.1. Ligand Preparation

Four pharmacologically active compounds of the OS plant, namely rosmarinic acid, sinensetin, eupatorin, and TMF, whose chemical structures are provided in Supplementary Data (Figure S3), were selected according to their abundance in the OS plant leaves. In addition, these compounds are previously known for their diverse therapeutic effects, including neuroprotective effects [12,20]. Three-dimensional conformers of these compounds were retrieved from the NCBI PubChem Database (https://pubchem.ncbi.nlm.nih.gov/) (accessed on 7 August 2024). All four compounds were assigned Gasteiger charges using AutoDockTools 1.5.7 [21].

#### 4.4.2. Protein Preparation

The crystal structure of MAO-A (PDB ID: 2bxr) at resolution 3 Å was downloaded from the Protein Data Bank (www.rcsb.org) [22]. The protein was initially cleaned from water molecules and other small molecules using BIOVIA^®^ Discovery Studio^®^ Visualizer v25.1.0.24284.

#### 4.4.3. Molecular Docking Simulation

Docking simulations were conducted using AutoDock 4.2.6. Grid boxes were centered on the active sites based on the co-crystallized ligand, clorgyline [22]. The grid points were X = 20.608, Y = 2.25, and Z = 5.692. The cleaned protein structure was processed using AutoDockTools 1.5.7, where polar hydrogen atoms and Kollman charges were assigned [21]. The simulation was carried out with 100 Lamarckian genetic algorithm runs to confirm sufficient conformational sampling and reproducibility of binding poses [21]. To evaluate the reliability of the molecular docking, the RMSD value of the re-docked ligand was calculated relative to the co-crystallized ligand (clorgyline), which yielded a value of 1.45 Å, signifying a high degree of agreement with the experimental binding pose. The configurations that had the lowest energy of binding (LEB) and the highest number of clusters were chosen for additional analysis. The lower the LEB, the higher the binding affinity. Interactions were analyzed using BIOVIA^®^ Discovery Studio^®^ Visualizer v25.1.0.24284. Hydrogen bonds, which are the most persistent interactions, and hydrophobic interactions were clearly exemplified in the 3D and 2D representations of a ligand bonded with the surrounding amino acids of the target protein.

### 4.5. In Vitro MAO-A Inhibition Activity

The potential inhibitory effect of the OS leaf ethanolic extracts and the selected pharmacologically active compounds-EUP, RA, and SEN-on the human MAO-A enzyme was assessed by fluorometric assay in a black 96-well plate using the MOA-A Inhibitor Screening Kit (Abcam). To prepare the stock solution, the test compounds were dissolved in 10% DMSO. The analytical procedures were performed according to the manufacturer’s protocol (Abcam, ab284510, Cambridge, UK). The final concentrations of the samples were 19.53 to 312.5 µg/mL for the ethanolic extracts (95% and 50%); 4.54 to 72.63 µM EUP; 4.33 to 69.4 µM RA; and 4.20 to 67.15 µM SEN. Additionally, we used positive and negative control wells with 0.1 µM clorgyline and 0.1% DMSO, respectively. Standards and samples were tested in triplicate. The fluorescence signal was measured (ex 530 nm and em 585 nm) at 25 °C for 10–30 min. The percent of inhibition was calculated using Equation (1):

(1) $$\mathrm{Relative}\;\mathrm{inhibition}=\frac{{\mathrm{slope}}_{EC}-{\mathrm{slope}}_{S}}{{\mathrm{slope}}_{EC}}\times 100$$

where *EC* is the enzyme control and *S* is the sample.

### 4.6. In Vivo Experimental Design

**Figure 7:**
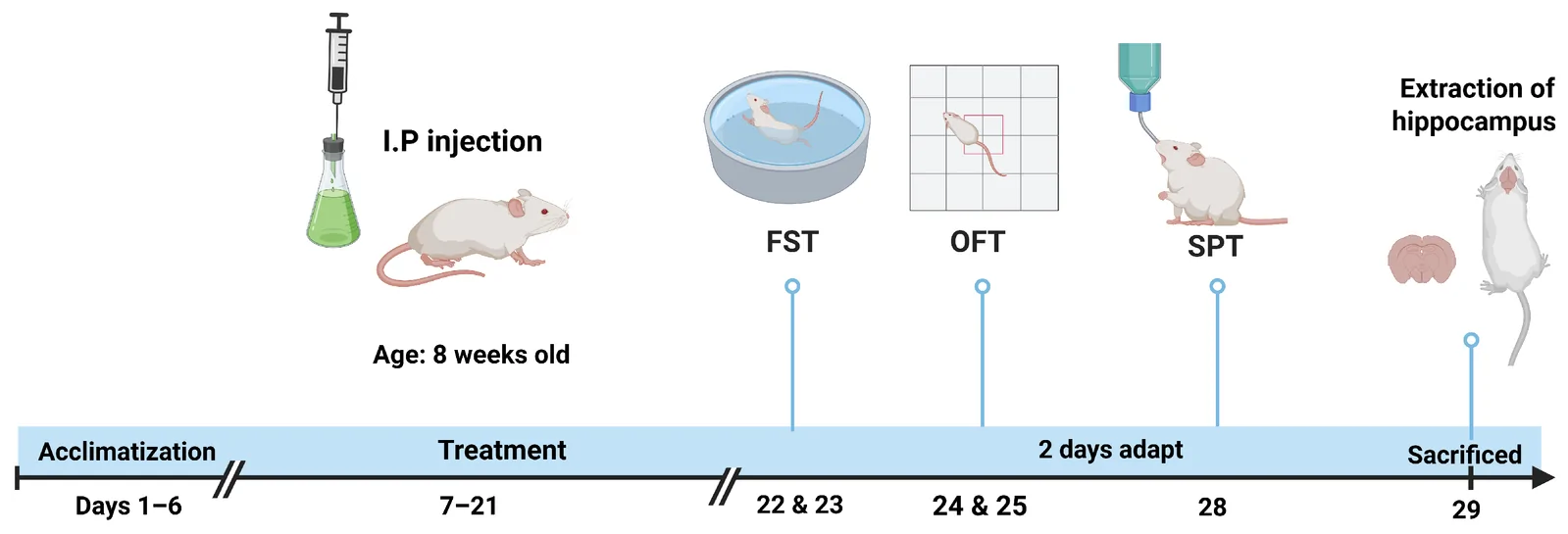
Schematic representation of timeline procedures for the assessment of antidepressant effects of *Orthosiphon stamineus* (OS) leaf ethanolic extract in rats. OFT, open field test; FST, forced swimming test; SPT, sucrose preference test.

The animals were divided into 6 groups. Each group consisted of 7 rats. The sample size for an independent means comparison was determined using G*Power version 3.1.9.7, assuming a Cohen’s d of 2.0 [23], α = 0.05, and power = 0.95 with an allocation ratio of 1:1. Reserpine was dissolved in a solution containing 1% DMSO and 2% Tween 80 at a dose of 0.2 mg/kg [24]. Fluoxetine hydrochloride was dissolved in 0.5% DMSO at a dose of 7 mg/kg [25]. The 95% ethanolic extract of OS leaves was dissolved in 0.5% DMSO and 2% Tween 80. Five of the groups were treated with reserpine (0.2 mg/kg), and four of these groups were additionally treated with either fluoxetine 7 mg/kg (as a positive control group) or OS extract at doses of 50, 100, or 150 mg/kg for 14 days before behavioral assessment. The sixth group served as a control group, which received the vehicle solution (0.5% DMSO and 2% Tween 80 solution) throughout the treatment period. Fluoxetine and OS leaf ethanolic extract were administered 1 h prior to reserpine administration. All the treatments were injected intraperitoneally. After 14 days of treatment, three behavioral tests were performed on the rats sequentially in the following order: open field test (OFT), forced swimming test (FST), and sucrose preference test (SPT). At the end of the experiment, the animals were sacrificed, and the brains of the animals were rapidly isolated for further biochemical and histological investigations. Figure 7 illustrates a schematic representation of the experimental design.

### 4.7. Animal Body Weight, Food and Water Consumption

The body weights of the animals were measured periodically throughout the treatment period. In addition, food and water intake was measured on days 1, 7, and 14. Intake was determined as the difference between the amount provided and the residual quantity at each time point.

### 4.8. Behavioral Tests

#### 4.8.1. Forced Swimming Test (FST)

The protocol outlined by Kraeuter and coworkers was utilized. On day 1 of the test, the rats were placed individually for 15 min in a container (60 cm height, 20 cm diameter) filled with 40 cm of water with a temperature of 21–23 °C. On day 2, the immobility time was measured during a 5 min session in which the rats remained floating without straining or moving [26].

#### 4.8.2. Open Field Test (OFT)

This test includes measurement of the animal locomotion, distance traveled, and time spent by animals exploring the central arena and time spent by animals near the walls (thigmotaxis). The session was conducted in low-light conditions, and the animal’s activity was monitored for 5 min using a 2.7.13 video tracking program (Pan Lab/Harvard Apparatus) [27]. The open field chamber is made up of an acrylic box with dimensions of W 40 cm × H 30 cm × L 40 cm. After each session, the chamber was washed with 70% ethanol. The software computes the time spent by the animals in the central arena compared to the periphery, locomotor activity, total distance traveled, velocity, and frequency of rearing.

#### 4.8.3. Sucrose Preference Test (SPT)

Anhedonia was evaluated by measuring the sucrose preference test. A decrease in the sucrose preference ratio compared to the control group is one of the characteristics of anhedonia. The sucrose preference test includes an evaluation of the preference of an animal between 2 bottles of sucrose and water. In this test, the animals were adapted to two drinking bottles. After acclimatization, the animals were isolated separately with two bottles of 1% sucrose and water for 24 h [28]. On the day of testing, the consumption (mL) of water and sucrose was calculated. The percentage of sucrose preference is measured as shown in Equation (2):

(2) $$\mathrm{Sucrose}\;\mathrm{Preference}\;(\%)=\frac{\mathrm{sucrose}\;\mathrm{solution}\;\mathrm{consumption}}{\mathrm{sucrose}\;\mathrm{solution}\;\mathrm{consumption}+\mathrm{water}\;\mathrm{consumption}}\times 100\%$$

### 4.9. Collection and Preparation of Samples

Twenty-four hours after the final behavioral test, the rats were anesthetized with an intraperitoneal injection of ketamine + xylazine cocktail (90 + 7 mg/kg) [29], before scarification. The entire brain of 3 out of 7 animals in each group was immersed in 10% buffered formalin for 24 h for fixation and processing before being embedded in paraffin using the Leica EG1160 Tissue Embedding Station (Leica Biosystems, Nussloch, Germany). The paraffin-embedded tissues were sliced with 4 µm thickness using an automated rotary microtome (HistoCore AUTOCUT^®^, Leica Biosystems, Germany), adhered to the normal glass slides for hematoxylin and eosin (H & E) staining and Superfrost slides for immunohistochemistry (IHC) staining, and then dried overnight at 37 °C. In contrast, the hippocampus of the remaining animals was promptly extracted and frozen at −80 °C for later investigation using the enzyme-linked immunosorbent assay (ELISA) technique.

### 4.10. Enzyme-Linked Immunosorbent Assay (ELISA)

The level of NE and 5-HT markers in the hippocampus was measured by competitive ELISA, while BDNF was measured by sandwich ELISA, according to the manufacturer’s instructions (Elabscience Biotechnology Inc., Houston, TX, USA). Briefly, about 0.1 g of rat hippocampus was homogenized with normal saline (100 mg/mL) at 25 Hz for 30 to 60 s on ice using the Up100H Ultrasonic Homogenizer (Hielscher Ultrasonics GmbH, Teltow, Germany), centrifuged at 10,000× *g* for 10 min at 4 °C, and the supernatant was kept at −80 °C before the ELISA test. A BCA colorimetric assay kit (Cat. No. E-BC-K318-M; Elabscience, Houston, TX, USA) was used to quantify the total protein concentration. For the quantification of NE and 5-HT, each pre-coated micro-ELISA plate (96-well plate) received 50 µL of diluted standards, samples, and blanks, each added in triplicate, followed by 50 µL of biotinylated detection antibody and incubated for 45 min at 37 °C. After being washed three times, HRP conjugate and substrate reagents were sequentially added, incubated, and stopped with a stop solution. For BDNF quantification, 100 µL of standards, samples, or blanks were incubated at 37 °C for 90 min before adding 100 µL of biotinylated detection antibody, HRP conjugate, and substrate reagent in separate steps, with incubation and washes between. The quantification of markers was determined at 450 nm absorbance. A four-parametric logistic (4PL) curve was plotted on a semi-log axis to calculate the concentration of the samples.

### 4.11. Hematoxylin–Eosin Staining (H&E)

Three rats from each group were examined with H&E staining using Leica ST5010 Autostainer XL (Leica Biosystems, Nussloch, Germany). The tissue sections were deparaffinized with xylene and rehydrated with graded alcohol. The slides were soaked with hematoxylin for 15 min and then differentiated with 1% hydrochloric acid in ethanol. Then, the slides were immersed in eosin staining for 2 min, dehydrated, and mounted in DPX solution to preserve the stained tissues for long-term analysis [30]. The stained slides were viewed under an Olympus BX51TRF light microscope (Olympus Corporation, Tokyo, Japan) at various magnifications to examine tissue morphology, cellular organization, and pathological changes.

### 4.12. Immunohistochemistry Assay

After fixation, the hippocampal slices were dewaxed and rehydrated by xylene, then rehydrated by a graded series of decreasing ethanol concentrations. The heat-induced epitope retrieval (HIER) method was used for antigen retrieval using Tris-EDTA buffer pH 9.0 from Sigma Aldrich (Petaling Jaya, Malaysia). The sections were blocked for 10 min at room temperature by peroxidase-blocking solution (Envision™ Detection Kit K5007) and then incubated for 15 min in 2% BSA in the wash buffer (1x Tris-buffered saline [TBS] + 0.5% Triton X-100 [Sigma Aldrich]). Diluted primary antibodies (rabbit polyclonal noradrenaline from Abbexa Ltd. (Cambridge, UK), rabbit polyclonal serotonin from Bio-Rad AbD Serotec Ltd. (Kidlington, UK), and rabbit polyclonal BDNF from Elabscience (Houston, TX, USA) were incubated overnight at 4 °C. Sections were washed with wash buffer three times for 5 min each. Afterwards, the sections were incubated with Envision + dual Link HRB (from the K5007 kit) for 30 min at room temperature. Visualization of antigen in tissue sections was detected by DAB (3,$3^{\prime }$-diaminobenzidine) staining for 10 min at room temperature. Finally, the sections were counterstained by hematoxylin for 30 s before dehydration and mounting with dibutyl phthalate polystyrene xylene (DPX) solution. The immunolabeling was investigated using an Olympus BX51TRF light microscope, and positive neurons were quantified in hippocampal subregions such as the dentate gyrus (DG) and cornu ammonis regions (CA1, CA2, and CA3) using image analysis Cell Sense Standard Olympus software at 40× magnification (50 µm). The percent of positive neurons is calculated according to the following formula:

(3) $$\mathrm{Positive}\;\mathrm{Neurons}=\frac{\mathrm{Number}\;\mathrm{of}\;\mathrm{Positive}\;\mathrm{Neurons}}{\mathrm{Total}\;\mathrm{Number}\;\mathrm{of}\;\mathrm{Neurons}}\times 100$$

### 4.13. Statistical Analysis

Data analysis was conducted using GraphPad Prism software (version 8.0.0, San Diego, CA, USA). For the in vitro analysis, ordinary one-way ANOVA, followed by Dunnett’s multiple comparisons test, was used to assess differences between treatment groups. IC50 was calculated using non-linear regression (4-parameter logistic curve) analysis. For the in vivo experiments, body weight, food, and water consumption during the treatment period were calculated using repeated measures of two-way ANOVA. The behavioral test data, ELISA, and IHC experiments were calculated using one-way ANOVA followed by the Bonferroni multiple comparison test. The data are expressed as the mean ± S.E.M.; a *p*-value of <0.05 was considered statistically significant.

## 5. Conclusions

Overall, this study demonstrates that the 95% ethanol extract of *Orthosiphon stamineus* leaves exerted a significant antidepressant-like effect in reserpine-induced depression in rats, which was likely mediated by monoaminergic modulation and enhanced BDNF expression, and it may partially involve MAO-A inhibition. However, the up-regulation of BDNF cannot establish a causal relationship with behavioral amelioration because TrkB activation and CREB phosphorylation were not evaluated in this study. Rosmarinic acid (RA) and eupatorin (EUP) may be responsible for observed effects, even though their individual contribution remains to be established experimentally. Thus, these findings suggest that the 95% ethanol extract of OS leaves could be a promising antidepressant agent; however, further studies are needed to directly measure brain MAO-A activity, elucidate the relevant molecular mechanisms, and investigate the relative contribution and possible synergistic interactions of individual active compounds of OS.

## Data Availability

The data supporting the findings of this study are available within the article and its Supplementary Materials files.

## Supplementary Materials

The following supporting information can be downloaded at: https://www.mdpi.com/article/10.3390/molecules31162892/s1, Table S1: Receptor interactions and lowest energies of binding of selected *Orthosiphon stamineus* (OS) components and clorgyline to the active site of the MAO-A enzyme. Figure S1: Three-dimensional (3D) and two-dimensional (2D) illustrations of compound configurations at the binding pocket of the MAO-A enzyme. (A) Clorgyline, (B) Eupatorin, (C) Rosmarinic acid, (D) Sinensetin, and (E) 3-hydroxy-5,6,7,4-tetramethoxyflavone (TMF). Figure S2: Representative photomicrographs of immunohistochemical staining in the rat hippocampus (40× magnification) for (a) 5-HT, (b) NE, and (c) BDNF across different treatment groups. A: Control; B: Reserpine; C: Fluoxetine; D: OS extract 50 mg/kg; E: OS extract 100 mg/kg; F: OS extract 150 mg/kg. Figure S3: Chemical structures of (A) eupatorin, (B) rosmarinic acid, (C) sinensetin, and (D) 3’-hydroxy-5,6,7,4’-tetramethoxyflavone (TMF), the principal pharmacological active compounds of *Orthosiphon stamineus* leaf.
