# Supplementary material for: Antidepressant-like Effects of Orthosiphon stamineus: Integrated Evidence from In Silico, In Vitro, and a Reserpine-Induced Depression Model
Source: Molecules. 2026 Aug 19;31(16):2892. doi: 10.3390/molecules31162892 (PMC13515085; doi:10.3390/molecules31162892)
Supplement: Supplementary file 1 [file molecules-31-02892-s001.zip › molecules-4485522-supplementary.pdf]

SUPPLEMENTARY DATA

INDEX

Table S1	Receptor interactions and lowest energies of binding of selected *Orthosiphon stamineus* (OS) components and clorgyline to the active site of the MAO-A enzyme.	-----------	2
Figure S1	Three-dimensional (3D) and two-dimensional (2D) illustrations of compound configurations at the binding pocket of the MAO-A enzyme. (A) Clorgyline, (B) Eupatorin, (C) Rosmarinic acid, (D) Sinensetin, and (E) 3-hydroxy-5,6,7,4-tetramethoxyflavone (TMF).	-----------	3
Figure S2	Representative photomicrographs of immunohistochemical staining in the rat hippocampus (40× magnification) for (a) 5-HT, (b) NE, and (c) BDNF across different treatment groups. A: Control; B: Reserpine; C: Fluoxetine; D: OS extract 50 mg/kg; E: OS extract 100 mg/kg; F: OS extract 150 mg/kg.	-----------	5
Figure S3	Chemical structures of (A) eupatorin, (B) rosmarinic acid, (C) sinensetin, and (D) 3'-hydroxy-5,6,7,4'-tetramethoxyflavone (TMF), the principal pharmacological active compounds of *Orthosiphon stamineus* leaf	---------	6

Table S1. Receptor interactions and lowest energies of binding of selected *Orthosiphon stamineus* (OS) components and clorgyline to the active site of the MAO-A enzyme.

Compound	LEB (kcal mol^{-1})	K_i (μM)	No. of H-Bonds	Amino acids involved in the interaction	
Clorgyline	-6.92	22.11	0	H-bond	
				Additional interactions	Tyr444, Tyr407, Tyr69, Leu337, Phe208
Eupatorin	-8.03	1.29	2	H-bond	Tyr444, Thr336
				Additional interactions	Phe208, Tyr444, Tyr407, Ile335
Rosmarinic acid	-8.17	1.03	5	H-bond	Tyr444, Arg206, Ser209
				Additional interactions	Tyr69, Phe208, Trp441, Gly7
Sinensetin	-8.21	0.96	1	H-bond	Ser209
				Additional interactions	Tyr407, Phe208, Tyr444, Phe209, Leu337, Ile335
TMF	-9.16	0.19	3	H-bond	Arg206, Thr73, Pro72
				Additional interactions	Tyr69, Pro72, Trp441, Phe352, Arg206, Phe208, Tyr444

TMF, 3-hydroxy-5,6,7,4-tetramethoxyflavone

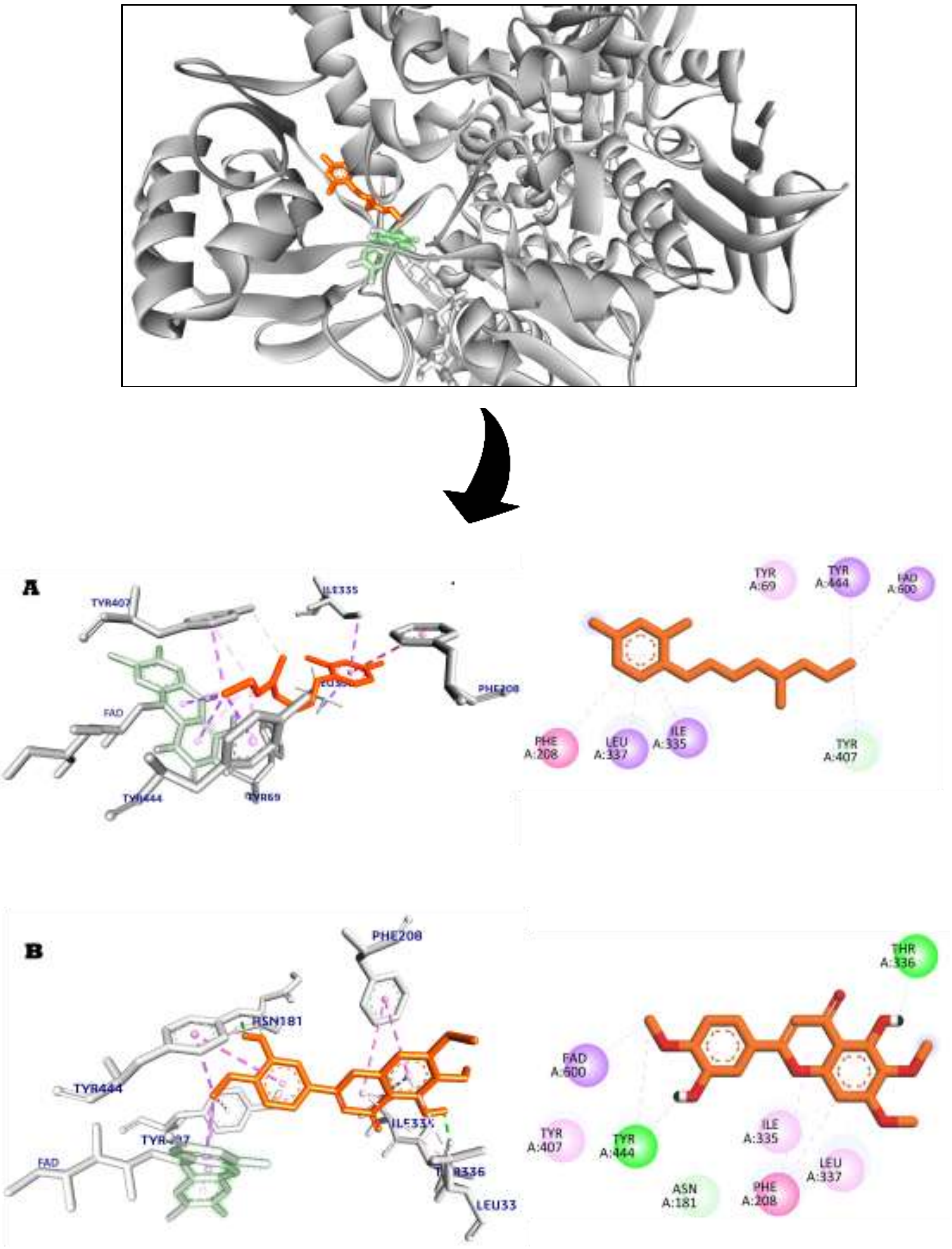
A
TYR407
ILE335
PHE208
FAD
TYR444
TYR69
TYR A:69
TYR A:444
FAD A:600
PHE A:208
LEU A:337
ILE A:335
TYR A:407
B
PHE208
ASN181
TYR444
FAD
THR A:336
FAD A:600
TYR A:407
TYR A:444
ILE A:335
LEU A:337
ASN A:181
PHE A:208

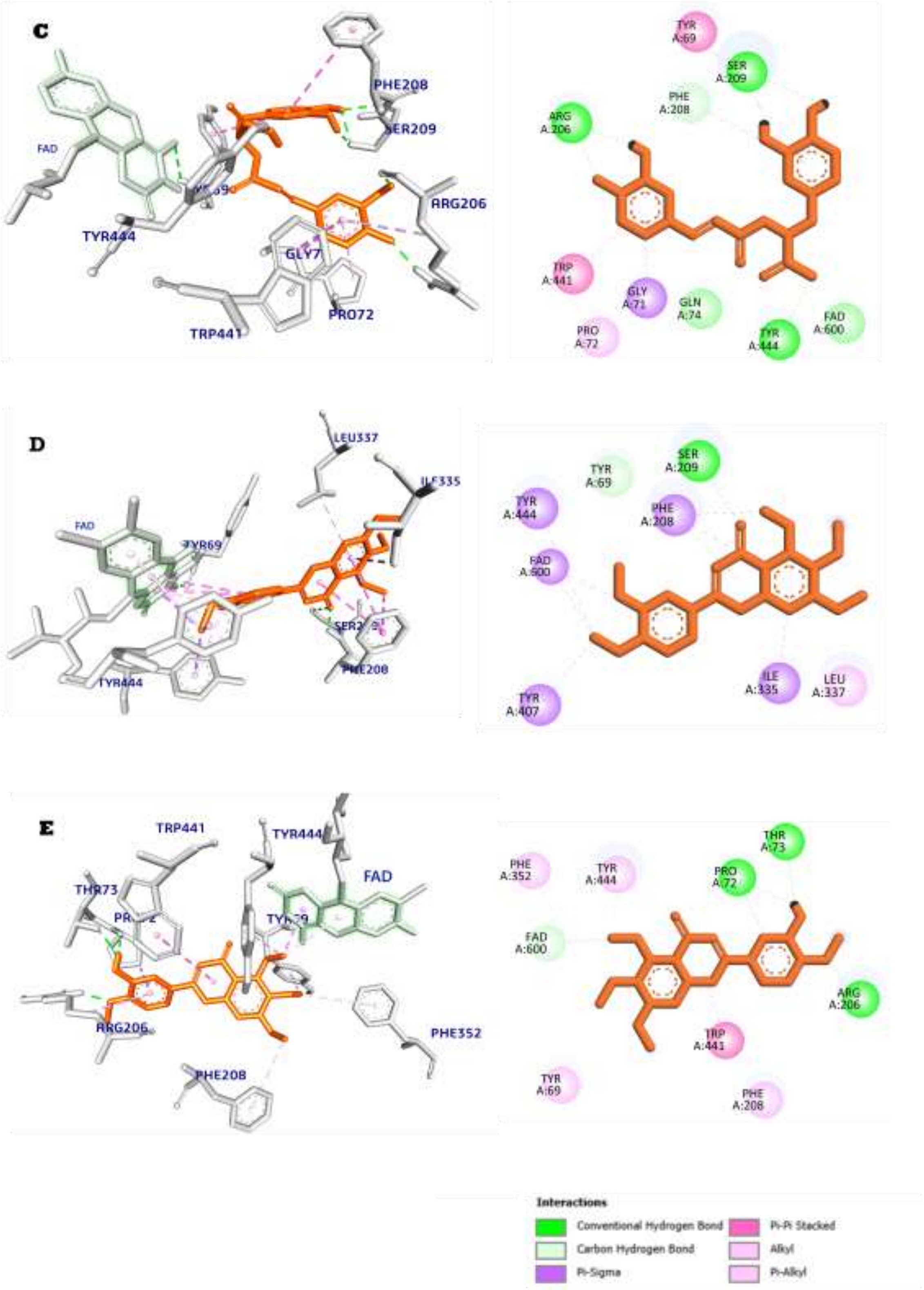

Figure S1. Three-dimensional (3D) and two-dimensional (2D) illustrations of compound configurations at the binding pocket of the MAO-A enzyme. (A) Clorgyline, (B) Eupatorin, (C) Rosmarinic acid, (D) Sinensetin, and (E) 3-hydroxy-5,6,7,4-tetramethoxyflavone (TMF).

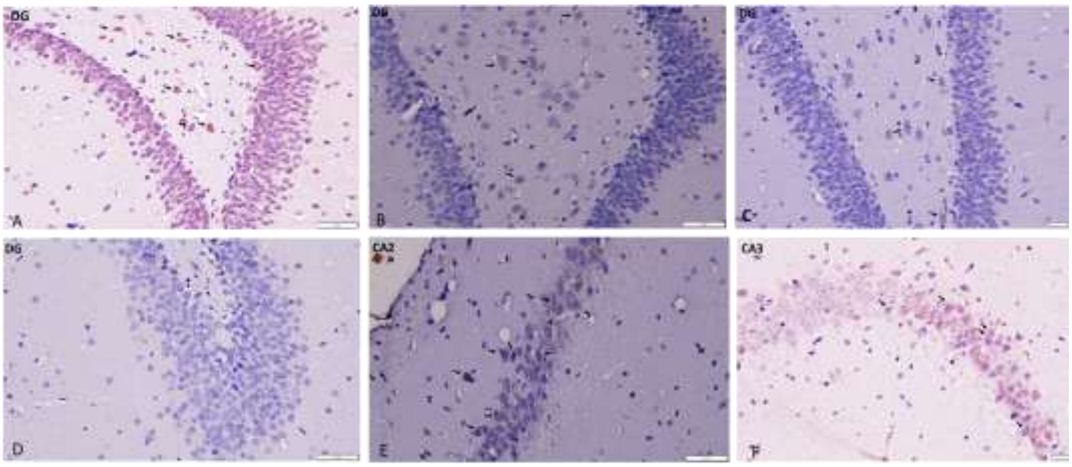

(a)

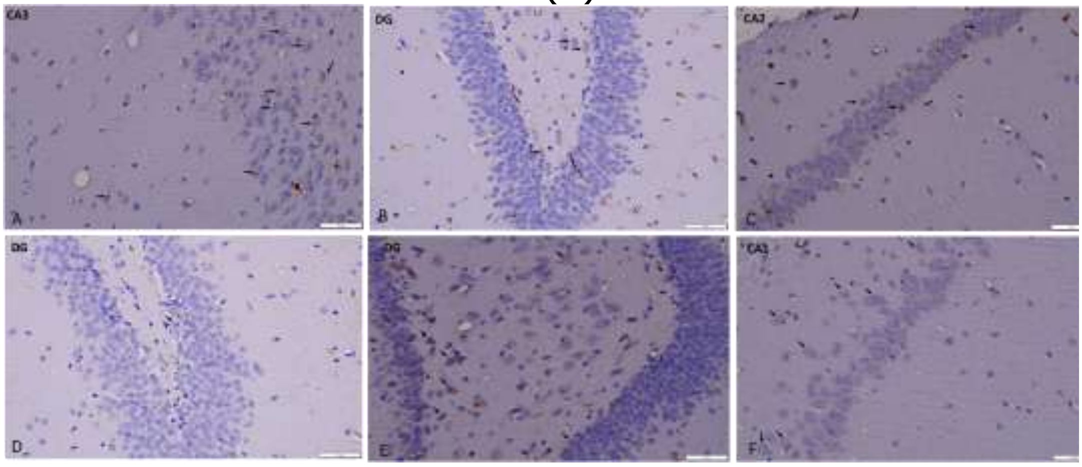

(b)

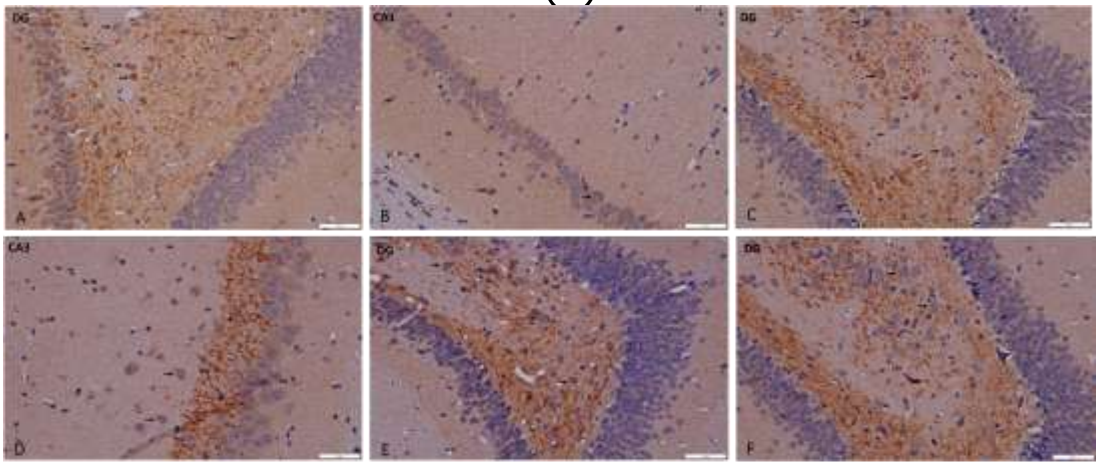

(c)

Figure S2. Representative photomicrographs of immunohistochemical staining in the rat hippocampus (40× magnification) for (a) 5-HT, (b) NE, and (c) BDNF across different treatment groups. A: Control; B: Reserpine; C: Fluoxetine; D: OS extract 50 mg/kg; E: OS extract 100 mg/kg; F: OS extract 150 mg/kg.

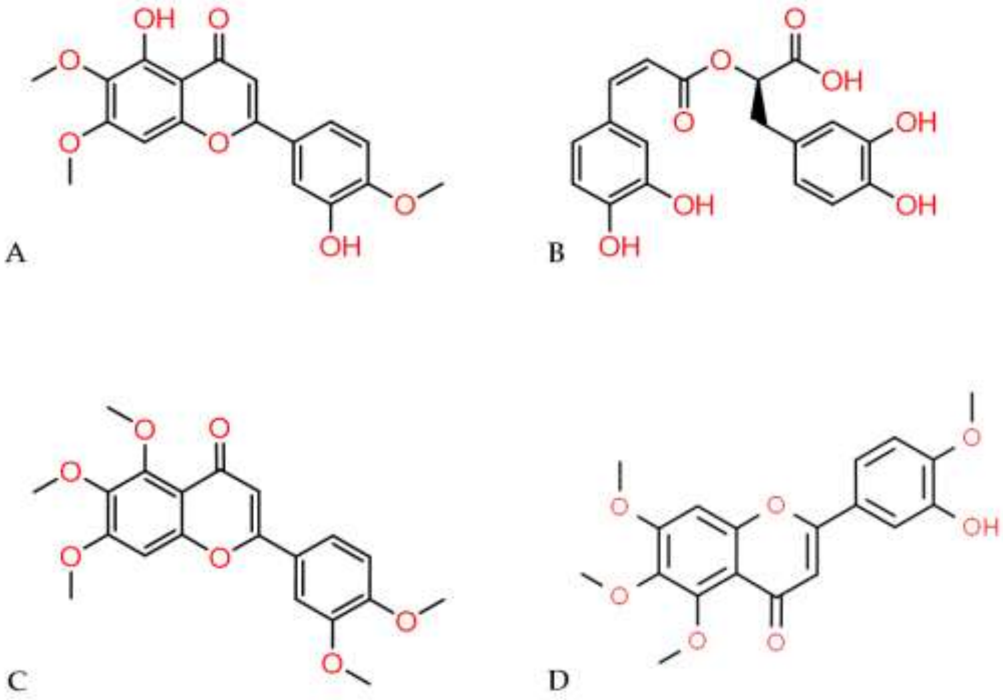

Figure S3. Chemical structures of (A) eupatorin, (B) rosmarinic acid, (C) sinensetin, and (D) 3'-hydroxy-5,6,7,4'-tetramethoxyflavone (TMF), the principal pharmacological active compounds of *Orthosiphon stamineus* leaf.
